# Open Characterization of Vaping Liquids in Canada: Chemical Profiles and Trends

**DOI:** 10.3389/fchem.2021.756716

**Published:** 2021-10-14

**Authors:** Ivana Kosarac, Cariton Kubwabo, Xinghua Fan, Shabana Siddique, Dora Petraccone, Wei He, Jun Man, Matthew Gagne, Kelly R. Thickett, Trevor K. Mischki

**Affiliations:** ^1^ Office of Research and Surveillance, Tobacco Control Directorate, Controlled Substances and Cannabis Branch, Health Canada, Ottawa, ON, Canada; ^2^ Exposure and Biomonitoring Division, Environmental and Radiation Sciences Directorate, Healthy Environments and Consumer Safety Branch, Health Canada, Ottawa, ON, Canada; ^3^ Food Research Division, Food Directorate, Health Products and Food Branch, Health Canada, Ottawa, ON, Canada; ^4^ Hazard Methodology Division, Safe Environments Directorate, Healthy Environments and Consumer Safety Branch, Health Canada, Ottawa, ON, Canada

**Keywords:** vaping, nicotine, non-targeted analysis, E-cigarettes, gc ms, flavours, pods, vaping liquids

## Abstract

Currently, there is a lack of comprehensive data on the diversity of chemicals present in vaping liquids. To address this gap, a non-targeted analysis of 825 vaping liquids collected between 2017 and 2019 from Canadian retailers was conducted. Prior to mass spectrometry analysis, samples were diluted 1:500 v/v with methanol or acetonitrile. Chemical compound separation and analysis was carried out using gas chromatography and triple quadrupole mass spectrometry (GC-MS/MS) systems operated in the full scan mode and mass range of 35–450 m/z. Mass spectrum for each sample was obtained in electron ionization at 70 eV and processed. Non-targeted identification workflow included use of automated mass spectral deconvolution and identification system (AMDIS), where required, as well as a number of commercially available spectral libraries. In order to validate identities, an in-house database of expected compounds previously detected in vaping liquids was used along with genuine analytical standards for compounds of interest. This resulted in a dataset of over 1,500 unique detected chemicals. Approximately half of these chemical compounds were detected only once in a single product and not in multiple products analyzed. For any sample analyzed, on average, 40% of the chemical constituents appeared to have flavouring properties. The remainder were nicotine and related alkaloids, processing, degradation or indirect additives, natural extractives and compounds with unknown roles. Data published here from the project on the Open Characterization of vaping liquids is unique as it offers a detailed understanding of products’ flavour chemical profiles, the presence and frequency of chemicals of potential health concern, as well as trends and changes in products’ chemical complexity over a three-year period. Non-targeted chemical surveillance such as this present valuable tools to public health officials and researchers in responding to emergent issues such as vaping associated lung injury or informing chemical based strategies which may be aimed at addressing product safety or appeal.

## 1 Introduction

Nicotine containing vaping products are a less harmful source of nicotine for people who smoke and are unable to cease the use of traditional tobacco products such as combustible cigarettes (Government of Canada, 2020a). Vaping products are not free from harm, in fact, for people who do not smoke, inhalation of vaping aerosol represents an unnecessary source of exposure to chemicals of potential health concern. Vaping products are a highly varied (Office of the Surgeon General, 2016) class of consumer products that continue to rapidly evolve and exhibit dynamic changes in product design and performance. This lack of product homogeneity as well as high variability in product use behaviors are thought to be one of the main reasons for not more fully understanding the harms and benefits of vaping products. The chemical exposure profile depends on vaping device parameters and design, user behavior and vaping liquid chemical composition. Elucidating the chemical composition of vaping liquids informs not only on the product’s safety and health risks relative to smoking, it can also provide information on aspects of product appeal and addiction liability among the products studied.

Nearly all vaping products intended for use with nicotine contain a liquid made up of approximately 90% carrier solvents (humectants-propylene glycol and glycerol), 0–6% nicotine with the remainder comprised of flavouring agents, processing aids, contaminants and water. The chemical heterogeneity of the vaping products originates from the variability among flavouring and processing agents used and presence of contaminants and post-formulation chemical transformations due to product storage and ageing. The traditional approach to analyzing chemicals in products is through targeted chemical analysis, wherein known chemicals are examined using optimized laboratory methods. Data generated using these methods offer an important support for decisions and actions but are limited to the known chemical space for which reference standards exist. In comparison to traditional chemical analytical methods, non-targeted analysis (NTA) methods aim to discover and prioritize total chemical exposures from as many as possible sources of chemicals present in the products. These methods use advanced analytical equipment, chemical libraries, and software based workflows to handle large datasets and detect as many chemicals as possible, including those previously unknown or understudied. The main aim of our study is to create a foundational library of chemicals present in Canadian vaping products using data collected from an analysis of 825 vaping liquids. This work can be used to better understand health risks, appeal and addiction associated with vaping products. In the current report we outline the study design, details of the non-targeted approach applied, large dataset organization and preliminary data analysis.

## 2 Materials and Methods

### 2.1 Chemicals and Reagents

99.7% pure propylene glycol and 99.2% pure glycerol were purchased from Sigma-Aldrich (Oakville, ON, Canada). HPLC grade methanol and acetonitrile were purchased from Fisher Scientific (Ottawa, ON, Canada). For a full list of individual compounds used to detect select chemicals refer to Supplementary Table S1.

### 2.2 Samples

A diverse sample of 825 vaping liquids were collected from vaping stores and physical retailers in seven cities across Canada and from online Canadian retailers, between 2017 and 2019. The samples included liquids of various nicotine concentrations (0–59 mg/ml) as well as varying proportions of propylene glycol (PG) and vegetable glycerine (VG) (0/100% to 100/0% PG/VG). Overall, samples represented 182 different brands. While 8% of samples had no declared product origin, a majority of products were formulated in Canada (82.5%), followed by United States (7.3%), and elsewhere (2.2%). Ninety-seven percent of products collected were packaged in refillable bottle format (30 or 60 ml, glass or plastic), while the rest were in plastic pod based format.

#### 2.2.1 Vaping Liquid Flavour Classification

Flavour–related information from product packaging and from product descriptions on manufacturer websites were used to inform the primary, intended flavour of the vaping liquid and systematically classify each sample into one of 18 flavour categories in a modified vaping liquid flavour wheel (Krüsemann et al., 2019), adapted for vaping liquid flavours available in the Canadian market. The following 18 flavour categories were used for product classification: Fruit (*N* = 108), Desserts (*N* = 76), Tobacco (*N* = 134), Mint/menthol (*N* = 97), Coffee (*N* = 33), Tea (*N* = 35), Energy Drinks (*N* = 19), Confectionary (*N* = 49), Savoury (*N* = 24), Spices (*N* = 19), Herbal/floral (*N* = 7), Nuts (*N* = 21), Alcohol (*N* = 34), Breakfast cereals (*N* = 33), Soft drinks (*N* = 29), Milk/cream/yogurt (*N* = 26), Unflavoured (*N* = 26), and Other (*N* = 55).

### 2.3 Sample Preparation

Following thorough sample mixing, 40 µl of each vaping liquid was diluted to 20 ml with methanol (Quantum TSQ GC MS/MS methodology) or acetonitrile (7000C GC MS/MS methodology). Diluted samples were vortex mixed and 1 µl was injected and analyzed using gas chromatography mass spectrometry. Solvent blank (methanol or acetonitrile) was injected after each sample to ensure no carryover between samples. Matrix blank consisting of propylene glycol and glycerol was used during the method development process to assess possibility of PG/VG thermal degradation during GC analysis.

### 2.4 GC MS/MS Analysis

Two instruments (Quantum TSQ and 7000C GC MS/MS) were used to acquire data, as such, two different methods were optimized. The acquisition mode for the both instruments was full-scan acquisition mode. The Quantum TSQ MS/MS instrument was coupled to a Trace GC Ultra gas chromatograph (Thermo Electron Corp.). The oven ramp for this instrument was set as followed: 65°C hold for 1 min, followed by an increase of 5°C/min to 280°C and held for 3 min thereafter. The source temperature and interface were held at 200°C and 250°C, respectively. The MS was operated in Electron Ionization, full-scan mode with scan range 35–450 m*/z* and emission current set at 100 µA. Source temperature was set to 200°C, while GC interface temperature was 250°C. The second instrument was a 6890N gas chromatograph coupled to a 7000C MSMS detector (Agilent Technologies Inc.). The GC oven programming was started at 50°C and held for 2 min, followed by a ramp at 5°C/min to 240°C where it was held for 3 min. Both source and the interface temperature were held at 280°C. The MS was operated in a full-scan acquisition mode and scan range 30–450 m*/z*. GC analyte separation was performed using the Zebron ZB-5HT GC capillary column (30 m × 0.25 mm × 0.25 µm) from Phenomenex (CA, United States) on both instruments. The injector temperature was set at 280°C for both GCs with splitless injection mode for GC Ultra and pulsed splitless mode for 6890N GC. In both cases GC carrier gas was helium operated in constant flow mode at 1 ml/min rate.

### 2.5 Non-Targeted Workflow

**FIGURE 1:**
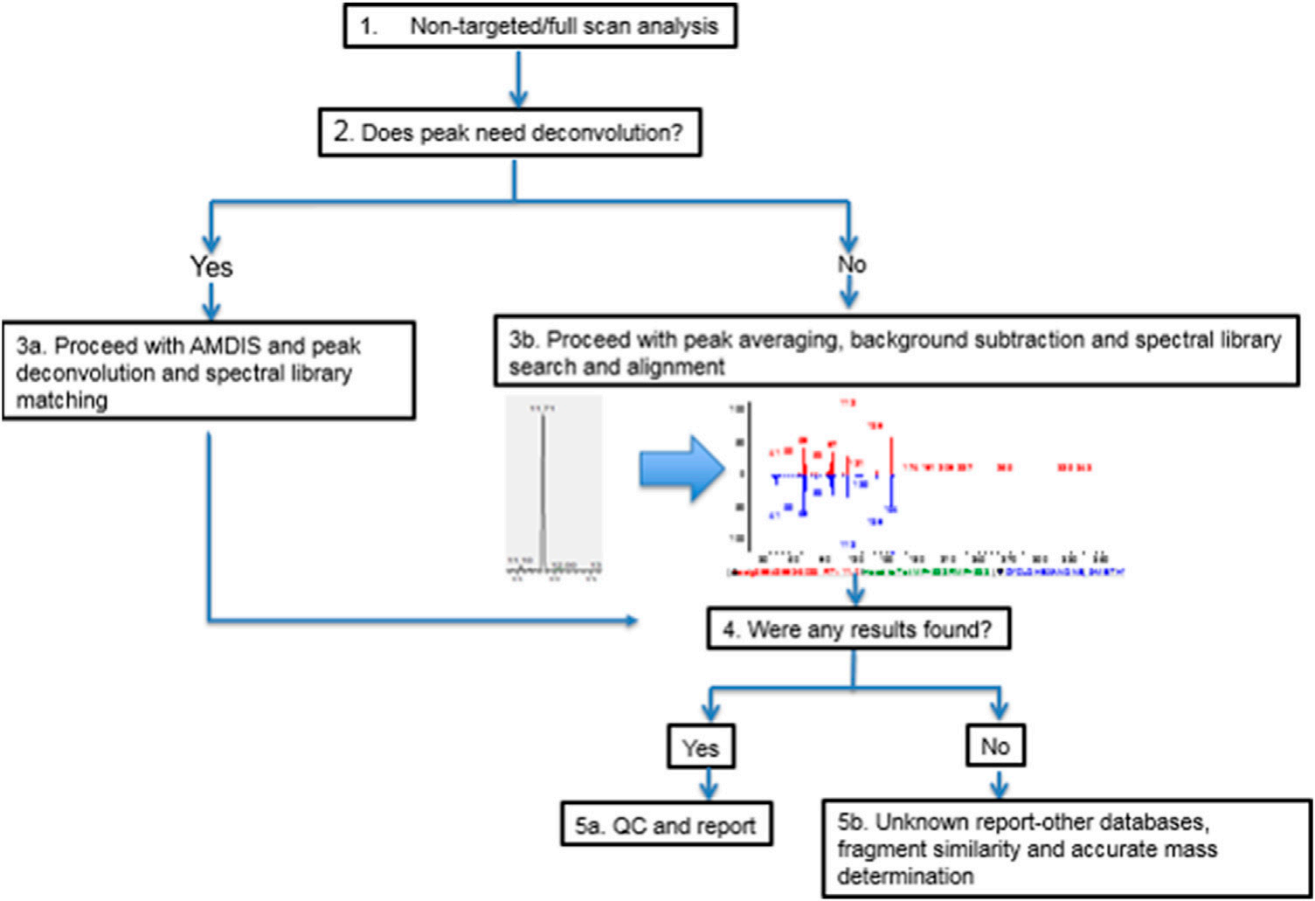
Non-targeted workflow.

Immediately following the sample analysis the chromatograms were processed as described in Figure 1. In some instances, where peak separation was poor, automated mass spectral deconvolution and identification system (AMDIS) (NIST, National Institute of Standards and Technology, 2019) was used for peak deconvolution. In general, the spectrum of individual compounds was matched against spectra from the National Institute of Standards and Technology (NIST 17) library reference peaks. In addition, the Agilent GC MS/MS instrument was also equipped with Wiley’s library of Mass Spectra of Flavors and Fragrances of Natural and Synthetic Compounds (FFNSC), 3rd Edition, while the Quantum GC also, used the Wiley Registry of Mass Spectral Data, 11th Edition for improved detection and confirmation. The peaks at signal intensity higher than signal to noise 3:1 are at first tentatively identified. In general, the compounds which score higher when matched against spectral libraries (>70 Agilent, >700 Quantum) and have an appropriate Retention Index, where available, are considered to be a good fit. In order to improve the analyte identification “starting confidence” or “prior probability” was utilized as previously described (Stein 2012). A database of previously detected and reported chemical compounds in vaping liquids from other published sources (*N* = 151, Supplementary Table S2 was used to develop categories of expected chemical compounds in vaping liquids (Table 1). Moreover, the same expected chemical compounds list served as the basis to set up an internal mass spectral database using genuine analytical standards of individual chemical compounds.

**TABLE 1:** Identification of detected chemicals.

Spectral library matching	Previously reported in vaping liquids (database)	
	Known (expected)	Unknown (unexpected)
Known	Known Known (e.g., nicotine)	Known Unknown (e.g., cinnamaldehyde propylene glycol acetal)
Unknown	Unknown Known (e.g., n-nitrosonornicotine-NNN)	Unknown Unknown

The chemical compounds with poor matching were compiled and a follow up analysis (e.g., accurate mass determination) will be performed in the future, if required.

### 2.6 Data Processing and Chemical Roles

Each identified chemical was assigned one or more roles in order to have a better understanding of the function they may have within a vaping liquid formulation. A literature synthesis was conducted which involved drawing from a variety of sources including published literature, open source websites and databases (e.g. PubChem (NIH, National Institutes of Health, 2021a), Chem Spider (Royal Society of Chemistry, 2021), The Human Metabolome Database (HMDB) (Wishart et al., 2018), Flavor DB (Garg et al., 2018), FooDB (Harrington et al., 2019)), manufacturer specifications, patents, Safety Data Sheets (SDS) and others, in order to aid in data processing and assignment of roles. Each chemical was classified into at least one of the six (6) roles: nicotine and related alkaloids, processing chemicals, natural extracts, flavours or fragrances, indirect additives and chemicals with unknown role. Supplementary information provides more information on specific functional role categories.

## 3 Results and Discussion

### 3.1 Workflow and Method Challenges

A number of challenges, which were successfully resolved, were encountered during this project. During the method development stages significant amount of time was invested in optimizing methodology as to minimize any compounds that may form during chemical analysis and degradation of product carrier solvents. More details and discussion are provided on method validation in Supplementary Section S2. Simple matrix blanks of PG and VG were put through dilution and analysis and no detected chemical compounds were formed during the analysis run time. Of note is that there was no carryover between samples analyzed as observed through testing of analytical blank samples between each injected sample. Simple dilution prior to mass spectrometry analysis did not result in any background contamination either. The 500 times solvent dilution often resulted in a broad glycerol peak and challenging chromatographic separation that, at times, would overlap with a signal for another chemical compound. In those instances, AMDIS was applied successfully, Supplementary Section S3. Processing of the resulting chromatograms was time consuming task, but was simplified using genuine analytical standards and established retention times for the group of chemical compounds previously reported to be present in vaping products (Table 1; Supplementary Table S1). This project was a significant undertaking (development of NTA methodologies and processing of large dataset with over 14,000 chemical compounds identified), it required diverse skillsets and frequent literature reviews to better elucidate chemical information such as functional groups and possible functional roles. While some parts of this process were automated, many steps still required manual quality control and review of results to ensure accuracy and completeness. Searching for individual chemical characteristics was done using Chemical Abstracts Services (CAS number) as provided in the mass spectral libraries. Significant data clean-up was performed in order to remove duplicate CAS numbers as some compounds may have multiple CAS numbers (e.g. menthol) and different mass spectral libraries may have preferences for CAS number provided as primary one.

### 3.2 Chemical Space

The actual chemical space of all products tested was 1,507 unique chemical compounds. Since some chemical compounds were detected in more than one product, total number of chemicals detected in 825 samples was over 14,000. Close to 50% (734/1,507) of all chemicals were detected in just one vaping liquid, illustrating the heterogeneity of this class of consumer products and infrequency of occurrence among chemical compounds used. Only four chemical compounds were detected in over 50% of all products studied. These include nicotine, the carrier solvents propylene glycol and glycerol, as well as β-Nicotyrine, a nicotine oxidation by-product that may form during storage (Wada et al., 1959). Seven hundred and thirty-eight products were labelled as nicotine-containing, however, among these products 14 were found not to contain any detectable nicotine. The lack of detection of nicotine in these samples was not due to the sensitivity of analytical method as this scan method is able to detect nicotine down to 0.03 mg/ml. The majority of samples with this discrepancy were, in fact, labelled to contain nicotine at over 9 mg/ml. Out of 87 products labelled as nicotine free, one product was detected to contain nicotine. These discrepancies on nicotine presence are likely due to poor manufacturing practices or lack of nicotine stability, as noted elsewhere (Goniewicz et al., 2015; Kavvalakis et al., 2015). Of note is that all samples in question were collected prior to September 2018 and, when labelled, were marked as manufactured prior to this date. These products likely precede the Government of Canada’s Tobacco and Vaping Products Act (Government of Canada, 2018b) which includes limits on nicotine concentrations and brings forward compliance and enforcement of the same.

**FIGURE 2:**
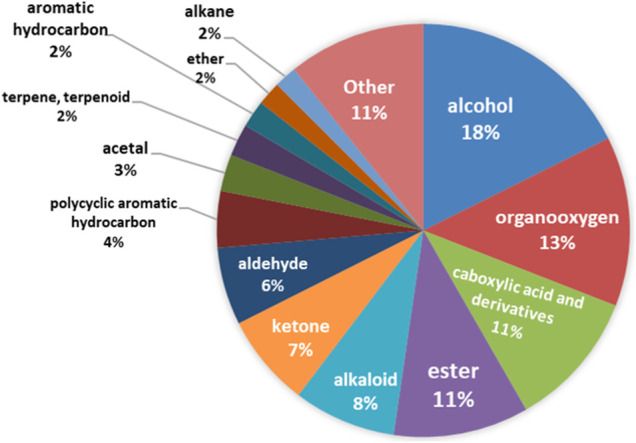
Detected chemical classes.

All chemical compounds detected in the course of the study can be classified into one of 170 chemical classes. The most frequently detected chemical classes are alcohol, organooxygen, carboxylic acid and derivatives, and esters, Figure 2.

### 3.3 Chemical Roles

**FIGURE 3:**
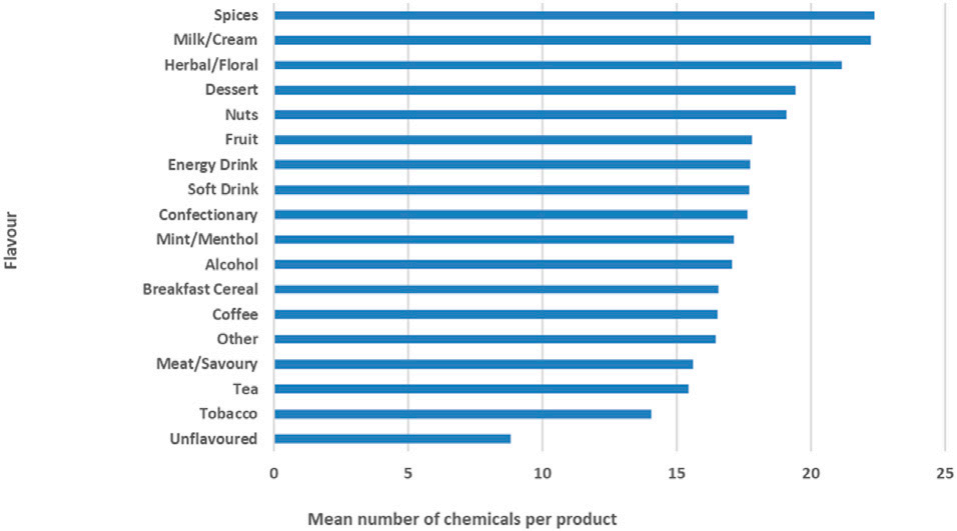
Number of detected chemicals per flavour category.

There were 87 (0.6%) chemical compounds for which it was not possible to assign or determine their identity using the mass spectral libraries available. In the future, samples with these compounds may be analyzed using different analytical approaches to identify them. Each chemical compound with a known identity was assigned at least one of the six functional roles using the various sources of peer-reviewed literature and supporting materials. Although identity was determined for the vast majority of detected chemicals, a functional role was not assigned to 8% of the chemicals detected as no supporting materials were found. Of note, a larger number of the chemicals with unknown roles have been previously detected in yeast (University of Washington, 2018). At this time, it is not known what the exact role or origin of yeast related chemicals in vaping liquids is. Autolyzed yeast extract is used as a flavour enhancer in foods and beverages (U.S. Food and Drug Administration, 2010; U.S. Food and Drug Administration, 2010), while microbial contamination of vaping products has been reported previously (Lee et al., 2019). Six percent of all chemicals were assigned indirect additive roles with supporting materials (Food and Drug Administration, 2017) often found among records on indirect additives on foods or food contact materials. It is likely these are found in products as a result of leaching into the vaping liquid during processing or packaging. Alkaloid roles were assigned to 10% of chemicals, which in the majority of cases included nicotine and related minor alkaloids. Thirteen percent of chemicals were found to have the natural extract role while 27% of chemicals were likely used as processing chemicals in the formulation. Examples of processing roles include emulsifiers, humectants, diluents and others. Forty-three percent of all chemicals detected were assigned a flavour or fragrance role. The number of individual chemicals per vaping liquid sample ranged between 4 and 66 compounds with a mean of 18 chemical compounds detected per product. Although a lower number of nicotine-salt based products were analyzed (*N* = 116) when compared to free-base nicotine products (*N* = 623), nicotine-salt products were found to contain a lower number of chemicals, with a mean of 16 chemicals detected per product. The number of chemical substances present in vaping liquids (e-liquids) can be used as one of the indicators of potential toxicity of the product, as reported previously by the group of researchers from North Carolina (Sassano et al., 2018) who concluded that increasing chemical numbers were associated with increasing toxicity when compared to solvent (PG/VG) vehicle in high-throughput *in-vitro* toxicity testing. In addition to nicotine type used in the product, the number of chemicals detected varied with the liquid’ flavour categories, Figure 3.

As expected, the unflavoured products appeared to have the least complex chemical profiles (mean number of nine chemicals), followed by the tobacco flavour category (mean number of 14 chemicals). The most complex chemical profiles were found in the categories of milk/cream (e.g., Yogurt) and spices (e.g. cinnamon), each with a mean of 22 detected chemicals. On average per product flavour category, the unflavoured category had the lowest proportion of flavour chemicals (15% of total chemicals), and energy drinks had the highest proportion of flavour chemicals (58% of total chemicals). Flavour categories such as fruit, confectionary and dessert, which may have a higher preference among youth, had higher proportions of flavour chemicals on average (48, 54 and 55% of all chemicals, respectively). This proportion of flavour compounds is somewhat lower compared to proportions (63%) reported by the Dutch study from European vaping products (Krüsemann et al., 2021). The differences could be due to the origins of the chemical datasets, as Dutch data is based on a reporting system where manufacturers provide information on ingredients added, while the non-targeted analysis based dataset results from chemical analysis which may detect impurities, indirect additives, as well as compounds that result from chemical reactions post-product formulation and product ageing (degradation, leaching and transformations). These additional compounds would increase the total number of compounds known to be present in the product, thereby decreasing the percentage of flavouring compound in the final composition.

**FIGURE 4:**
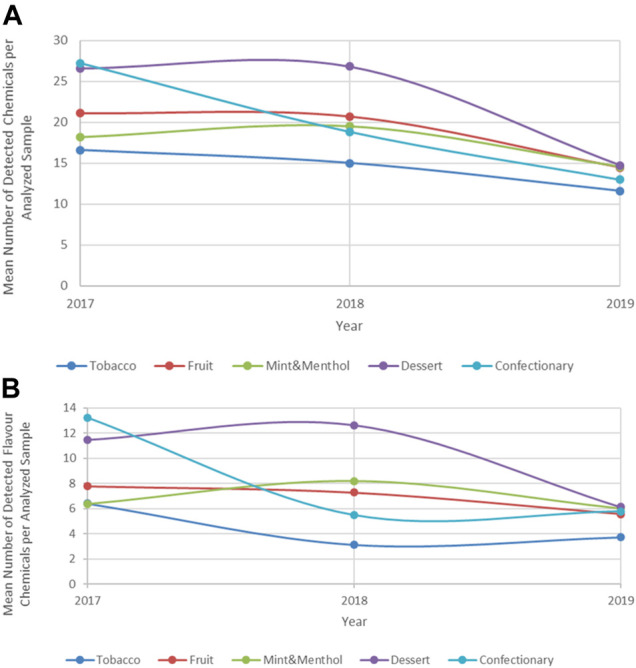
Overall chemicals **(A)** and Flavour chemicals **(B)** per popular flavour category, 2017–2019.

Of note is that the mean number of chemicals detected per product has in fact changed over the years; products collected in 2017 and 2018 appear to have a significantly higher number of chemical compounds when compared to those collected in 2019. This trend is observed regardless of flavour category analyzed, Figure 4A. When the trend is examined for the number of flavour compounds over this time period and in the same products a similar trend emerges, suggesting a decrease in the chemical flavour complexities among this group of products, Figure 4B.

This trend could be in part explained by the higher frequency of nicotine-salt based products post 2018 which on average appear to contain a lower number of chemicals. Nicotine-salts are perceived to provide a less harsh and smoother sensory experience for the product users (Leventhal et al., 2021), thus it is likely they require less flavouring agents to mask the sensory experience normally associated with free-base nicotine products.

### 3.4 Flavour Chemicals of Concern

Vaping products on the Canadian market come in a variety of flavour categories. In the past few years, youth vaping prevalence has increased in Canada (Government of Canada, 2020a) and flavours play an important role in attracting youth to vaping products. Recent evidence suggests that youth prefer flavour categories such as fruit, confectionary and dessert (Government of Canada, 2018a; O’Connor et al., 2019). The chemicals detected in products are used to better understand flavour chemicals and their role in imparting intended or declared product flavours. Vaping product formulations are the manufacturer’s interpretation of the intended or declared flavour. Our data indicates that the chemical space of each flavour category is diverse and there is a high degree of chemical overlap between flavour categories. Similar to previously published studies (Tierney et al., 2016; Omaiye et al., 2019), our data shows that vaping liquids contain some of the same flavour chemicals despite their flavour category. Except for mint/menthol, herbal/floral and unflavoured category, across all other products, the top five most frequently detected flavour chemicals (Table 2) were vanillin, ethyl maltol, ethyl vanillin, vanillin propylene glycol acetal and cyclotene. Vanillin, ethyl maltol and ethyl vanillin were in the top five flavouring chemicals for more than half of the flavour categories studied. Collectively, the top five chemicals have flavour descriptors such as “sweet,” “creamy” and “vanilla” (Good Scents Company, 2021). Vanillin and ethyl maltol, but not ethyl vanillin, were the most frequently detected flavour chemical in the three categories likely to be more appealing to youth. Ethyl maltol is a sweetener, with a sweet, caramellic, jammy, strawberry-like odor description and sweet, burnt cotton candy, caramel-like taste. Perception of sweet flavour in vaping products has been shown to produce greater appeal and perceived sweetness ratings among young vapers (Goldenson et al., 2016). Moreover, sweet perception and appealing flavours can enhance nicotine reward reinforcing effects in vaping and other tobacco products (Kroemer et al., 2018; Patten and De Biasi 2020).

**TABLE 2:** The top five most frequently identified chemicals in all flavour categories and the flavour/odour description from the Good Scents Company website.

CAS	Chemical name	Organoleptic properties^a^	Avg % frequency (in all liquids)	# Flavour categories detected as a top 5 chemical	Top 5 chemical in fruit (F), confectionary (C); dessert (D)
121-33-5	Vanillin	Vanilla sweet creamy spicy phenolic milky	45	14	F, C, D
4940-11-8	Ethyl maltol	Sweet burnt sugar candy jam strawberry	30	12	F, C, D
121-32-4	Ethyl Vanillin	Sweet creamy vanilla smooth caramellic	30	11	C, D
68527-74-2	Vanillin propylene glycol acetal	Sweet vanilla creamy phenolic smoky powdery	19	6	D
80-71-7	Cyclotene	Caramellic maple	14	4	None

a The Good Scents Company Information System (Good Scents Company, 2021).

In published studies, concentrations of ethyl maltol in vaping liquids range between undetectable to 4,200 μg/ml (Aszyk et al., 2017; Behar et al., 2018), compared to average maximum concentration ranges of 12.4–152 μg/ml in non-alcoholic beverages and baked goods, respectively, on which Flavor Extract Manufacturers Association (FEMA, The Flavor and Extract Manufacturers Association of the United States, 2021) Expert Panel based its’ judgments that this substance is safe for ingestion (Oser and Ford 1977). Although generally recognized as safe for ingestion, the health effects of ethyl maltol, and more broadly the majority of flavour compounds, have not been assessed for the inhalation route (Flavor and Extract Manufacturers Association, 2021). Currently, published studies on vaping flavours focus on cytotoxic and mutagenic effects in cell models (Behar et al., 2018; Muthumalage et al., 2018); translating these study findings into a real-life setting is challenging. While inhalation toxicity data is scarce for some compounds, certain vaping flavour compounds are recognized as those of concern for human health. For example, diacetyl and 2,3 pentanedione are two buttery flavours, shown to cause lung and respiratory airways damage in animal models and are associated with respiratory disease and decreased lung function in occupationally exposed employees of food flavouring and food manufacturing facilities (NIOSH, The National Institute for Occupational Safety and Health, 2016). While diacetyl was detected in two vaping liquids acquired prior to 2018, 2,3 pentanedione was not detected in any vaping liquids analyzed in the Open Characterization dataset. Another flavour, the monoterpene pulegone typically found in extracts of mint oil, has been previously detected in vaping products (Hutzler et al., 2014; Geiss et al., 2015). This chemical has been shown to induce some carcinogenic effects in mice and rats (National Toxicology Program, 2011). In the Open Characterization analysis, 11 out of 825 (1.3%) products were found to contain pulegone at unknown concentration levels, mainly mint/menthol flavoured products (9/11 products). Currently, no evidence is available that pulegone has any vaping-related health effects in humans.

### 3.5 Chemicals of Health Concern

Within this dataset, the quantification of all chemicals identified is untenable given the targeted study method developments may take years to complete. Chemical prioritization or screening based on known hazards was used to develop a list of chemicals for quantification. Providing exposure estimates through targeted analytical studies focused on these prioritized chemicals will provide sufficient information to better elucidate the risk. The majority of studies provide results on relative risk and comparison to tobacco cigarettes. Vaping products in fact infrequently contain tobacco specific toxicants and even in cases when they do, these are often present at much lower concentrations as observed in the exposure studies on product users (Goniewicz et al., 2018; Engineering, and Medicine National Academies of Sciences, 2018). For example, in our study there was only one product that was found to contain N-Nitrosodimethylamine (NDMA); no other nitrosamines were detected. In addition to NDMA, 9 out of 93 US FDA’s Harmful and Potentially Harmful Constituents (HPHC) (FDA, US Food and Drug Administration, 2012) were detected in Open Characterization samples (Table 3).

**TABLE 3:** Established list of constituents identified by US FDA as harmful and potentially harmful constituents and their detection frequency in vaping liquids.

Constituent	CAS RN	Carcinogen (CA), respiratory toxicant (RT), cardiovascular toxicant (CT), reproductive or developmental toxicant (RDT), addictive (AD)	Frequency of detection (%)
Acetaldehyde	75-07-0	CA, RT, AD	1.2
Coumarin	91-64-5	Banned in food	0.6
Ethylbenzene	100-41-4	CA	4.5
Ethylene oxide	75-21-8	CA, RT, RDT	0.5
Methyl ethyl ketone	78-93-3	RT	1.0
Naphthalene	91-20-3	CA, RT	12.2
N-Nitrosodimethylamine (NDMA)	62-75-9	CA	0.1
Phenol	108-95-2	RT, CT	0.6
Quinoline	91-22-5	CA	0.5
Styrene	100-42-5	CA	0.7

The reasons behind the higher frequency of detection of naphthalene compared to other HPHC chemicals are unclear at this time; this Polycyclic Aromatic Hydrocarbon is normally present in tobacco smoke, but also in the extracts of various fruits and other plants (Gómez et al., 1993; Paris et al., 2018), so it is possible that naphthalene originates from the natural extracts used to flavour the products. Of note is that other methylated and naphthalene-related structural analogs, not on the HPHC list, were also detected in vaping products studied. For example, 1-methyl naphthalene, a flavour and fragrance agent normally found in fruits (Good Scents Company, 2021), is also detected in 12% of products analyzed. Exposure of laboratory animals to 1- and 2-methylnaphtalene resulted in spleen and organ damage while mice exposed dermally for 30 weeks developed pulmonary alveolar proteinosis. Humans exposed to this compound developed skin irritation and skin photosensitization (NIH, National Institutes of Health, 2021b). In 2019, USFDA proposed the addition of 19 chemical compounds to an existing HPHC list of 93 (Food and Drug Administration, 2017), mainly to reflect potentially harmful chemicals present in vaping products. The first proposed chemical is glycidol, a probable human carcinogen (International Agency for Research on Cancer, 2000) thought to result from thermal degradation of glycerol. Glycidol has been previously detected in vaping product emissions (Sleiman et al., 2016) and was found in 3% of the liquids tested. Non-targeted studies such as this provide datasets that can inform future steps and ultimately characterize product-use specific harms. The prioritization can consider chemicals with already established health effects of concern, detection frequency or chemical presence in products with high market share. In our dataset, most chemicals of concern were not detected in the majority (>50%) of products studied, indicating that the chemicals of concern can be used to identify products for which the ingredients used may be a cause for concern. The goal is to provide information that would lead to products which minimize the risk of vaping products for consumers looking to completely switch from combustible tobacco products.

In comparison to traditional chemical analytical methods, non-targeted analysis (NTA) methods aim to discover as many chemicals as possible in products, including those previously unknown or with limited data. To date, there has been only one published study using non-targeted screening of Canadian vaping liquids (Czoli et al., 2019). One hundred and sixty-six vaping liquids collected in 2015 were analyzed using a gas chromatography mass spectrometry instrument with limited sensitivity and resolution. Similarly, a U.S. dataset generated by the Centre for Tobacco Regulatory Science and Lung Health (Center for Tobacco Regulatory Science and Lung Health, 2021), chemically characterized approximately 300 vaping product samples; significantly fewer than the Canadian Open Characterization dataset (*N* = 825). Closed pod-system brands that make up a majority of the vaping market in Canada were not included in the U.S. dataset. In addition, limited information is available on the products tested in the U.S. including classification by flavour categories, as their product names are not self-explanatory (e.g. Carnage, Magic Dragon, etc.). Finally, it is unknown how many of these U.S. products are available for sale in Canada. These factors present challenges in comparing the two datasets. Overall, valuable information can be determined by evaluating different market datasets, however direct comparisons are challenging given the heterogeneity of vaping products within and between different regions.

## Data Availability

The original contributions presented in the study are included in the article/Supplementary Material, further inquiries can be directed to the corresponding author.
