# Supplementary material for: Open Characterization of Vaping Liquids in Canada: Chemical Profiles and Trends
Source: Front Chem. 2021 Oct 14;9:756716. doi: 10.3389/fchem.2021.756716 (PMC8551961; doi:10.3389/fchem.2021.756716)
Supplement: Supplementary file 1 [file DataSheet1.pdf]

Open Characterization of vaping liquids in Canada: chemical profiles and trends

Ivana Kosarac[1*], Cariton Kubwabo[2], Xinghua Fan[2], Shabana Siddique[2], Dora Petraccone[1], Wei He[2], Jun Man[3], Matthew Gagne[4], Kelly R. Thickett[1], Trevor K. Mischki[1]

[1] Office of Research and Surveillance, Tobacco Control Directorate, Controlled Substances and Cannabis Branch, Health Canada, Ottawa, ON, Canada

[2] Exposure and Biomonitoring Division, Environmental and Radiation Sciences Directorate, Healthy Environments and Consumer Safety Branch, Health Canada, Ottawa, ON, Canada

[3] Food Research Division, Food Directorate, Health Products and Food Branch, Health Canada, Ottawa, ON, Canada

[4] Hazard Methodology Division, Safe Environments Directorate, Healthy Environments and Consumer Safety Branch, Health Canada, Ottawa, ON, Canada

Supplementary Material

Table S1: Chemical compounds of analytical grade or higher purchased from Sigma Aldrich Canada.

**CASRN**	**Common Name**	**Chemical Name**
928-96-1	(3Z)-3-Hexen-1-ol	Leaf alcohol
115-95-7	1,6-octadien-3-ol, 3,7-dimethyl-, acetate	1,6-octadien-3-ol, 3,7-dimethyl-, acetate
71-41-0	1-amylalcohol	1-amylalcohol
123-92-2	1-butanol, 3-methyl-, acetate	1-butanol, 3-methyl-, acetate
23726-92-3	1-Damascone	1-Damascone
111-27-3	1-hexanol	1-hexanol
108-48-5	2,6 ethylpyridine	2,6 ethylpyridine
1124-11-4	2356-Tetramethylpyrazine	2356-Tetramethylpyrazine
1072-83-9	2-acetylpyrrole	2-acetylpyrrole

15679-19-3	2-isopropyl-4-methylthiazole	2-isopropyl-4-methylthiazole
35158-25-9	2-isopropyl-5-methyl-2-hexenal	2-isopropyl-5-methyl-2-hexenal
119-84-6	3,4-dihydrocoumarin	3,4-dihydrocoumarin
93-18-5	3-methyl-3-phenylglycidate	3-methyl-3-phenylglycidate
122-00-9	4-methylacetophenone	4-methylacetophenone
620-02-0	5-methylfurfural	5-methylfurfural
24851-98-7	Methyl dihydrojasmonate	methyl 2-(3-oxo-2-pentylcyclopentyl)acetate
10094-34-5	a,a-dimethylphenethyl butyrate	a,a-dimethylphenethyl butyrate
22047-25-2	acetylpyrazine	acetylpyrazine
127-41-3	a-ionone	Alpha-ionone
104-21-2	Anisyl acetate	Anisyl acetate
98-55-5	a-terpineol	a-terpineol
80450-69-7	cis-Hedione	cis-Hedione
100-52-7	Benzaldehyde	Benzaldehyde
2568-25-4	Benzaldehyde PG acetal	Benzaldehyde PG acetal
101-97-3	benzeneacetic acid, ethyl ester	benzeneacetic acid, ethyl ester
140-11-4	Benzyl acetate	Benzyl acetate
100-51-6	Benzyl alcohol	Benzyl alcohol
106-27-4	butanoic acid, 3-methylbutyl ester	butanoic acid, 3-methylbutyl ester
3681-71-8	Cis-3-hexenyl acetate	Cis-3-hexenyl acetate

35852-46-1	cis-3-hexenyli-valerate	cis-3-hexenyli-valerate
106-22-9	Citronellol	Citronellol
21834-92-4	Cocal	Cocal
80-71-7	Corylone	Corylone
112-31-2	Decanal	Decanal
105-53-3	Diethyl malonate	Diethyl malonate
123-25-1	Diethyl succinate	butanedioic acid, diethyl ester
89-78-1	DL-Menthol	DL-Menthol
1122-62-9	ethanone, 1-(2-pyridinyl)-	ethanone, 1-(2-pyridinyl)-
13327-56-5	Ethyl 3-(methylthio)propionate	Ethyl 3-(methylthio)propionate
141-97-9	Ethyl acetoacetate	Ethyl acetoacetate
103-36-6	Ethyl cinnamate	Ethyl cinnamate
106-30-9	Ethyl heptanoate	Ethyl heptanoate
123-66-0	Ethyl hexanoate	Ethyl hexanoate
97-64-3	Ethyl lactate	Ethyl lactate
4940-11-8	Ethyl maltol	2-Ethyl-3-hydroxy-4-pyrone
121-32-4	Ethyl vanillin	Ethyl vanillin
7452-79-1	Ethyl-2-methylbutyrate	butanoic acid, 2-methyl-, ethyl ester
97-53-0	Eugenol	Eugenol
3658-77-3	Furaneol	Furaneol

98-00-0	Furfuryl alcohol	Furfuryl alcohol
96-48-0	gamma-butyrolactone	gamma-butyrolactone
2305-05-7	g-dodecalactone	g-dodecalactone
105-90-8	Geranyl propionate	Geranyl propionate
695-06-7	g-hexalactone	g-hexalactone
56-81-5	Glycerol	Propane-1,2,3-triol
108-29-2	g-valeroactone	g-valeroactone
142-92-7	Hexyl acetate	Hexyl acetate
6378-65-0	Hexyl hexanoate	Hexyl hexanoate
659-70-1	Isoamyl isovalerate	Isoamyl isovalerate
6728-26-3	Leaf aldehyde	Leaf aldehyde
138-86-3	Limonene	Limonene
78-70-6	Linalool	Linalool
60047-17-8	Linalool oxide	Linalool oxide
79-20-9	L-menthyl acetate	L-menthyl acetate/menthyl acetate
118-71-8	Maltol	Maltol
106-72-9	Melonal	Melonal
1490-04-6	Menthol	Menthol
89-80-5	Menthone	Menthone
103-26-4	Methyl cinnamate	Methyl cinnamate

110-93-0	Methyl heptenone	Methyl heptenone
119-36-8	Methyl salicylate	Methyl salicylate
106-25-2	Nerol + geraniol	Nerol + geraniol
54-11-5	Nicotine	Nicotine
487-19-4	Nicotyrine	3-(1-methylpyrrol-2-yl)pyridine
140-26-1	Phenethyl isovalerate	Phenethyl isovalerate
57-55-6	Propane-1,2-diol	Propylene Glycol
123-32-0	Pyrazine, 2,5-dimethyl-	Pyrazine, 2,5-dimethyl-
109-08-0	Pyrazine, methyl-	Pyrazine, methyl-
110-86-1	pyridine	pyridine
5471-51-2	Raspberry ketone	Raspberry ketone
93-92-5	Styralyl acetate	Styralyl acetate
78-69-3	Tetrahydrolinalol	Tetrahydrolinalol
36431-72-8	Theaspirane	Theaspirane
928-95-0	trans-2-hexenol	(E)-2-Hexen-1-ol
14667-55-1	Trimethyl-pyrazine	pyrazine, trimethyl-
121-33-5	Vanillin	Vanillin
706-14-9	γ-Decalactone	γ-Decalactone
104-61-0	γ-Nonalactone	γ-Nonalactone
705-86-2	δ-decalactone	δ-decalactone

2721-22-4	δ-tetradecalactone	δ-tetradecalactone

Table S2 References used to develop a list of expected chemicals in vaping liquids

Ref ID	Reference Type	Authors, Primary	Periodical Abbrev	Pub Year	Volume	Issue	Start Page
1	Journal Article	Bahl,V. et al.	Reprod.Toxicol.	2012	34	4	529
2	Journal Article	Ballbe,M. et al.	Environ.Res.	2014	135		76
3	Journal Article	Barrington-Trimis,J. L.; et al.	JAMA	2014	312	23	2493
4	Journal Article	Behar,R. Z. et al.	Toxicol.In.Vitro.	2014	28	8	1521
5	Journal Article	Behar,R. Z. et al.	Toxicol.In.Vitro.	2014	28	2	198
6	Journal Article	Bekki,K. et al.	Int.J.Environ.Res.Public.Health.	2014	11	11	11192
7	Journal Article	Benowitz, N.L. et al.	JAMA	2013	310	7	685
8	Journal Article	Bertholon,J. F. et al.	Respiration	2013	86	5	433
9	Journal Article	Bhatnagar,A. et al.	Circulation	2014	130	16	1418
10	Journal Article	Burstyn,I.	BMC Public Health	2014	14		18
11	Journal Article	Cahn,Z. et al.	J.Public Health Policy	2011	32	1	16

12	Journal Article	Callahan-Lyon,P.	Tob.Control	2014			ii36
13	Journal Article	Cervellati ,F. et al.	Toxicol.In.Vitro .	2014	28	5	999
14	Journal Article	Chatham-Stephens, K. et al.	MMWR Morb.Mortal.W kly.Rep.	2014	63	13	292
15	Journal Article	Chen,I. L.	Nicotine Tob.Res.	2013	15	2	615
16	Journal Article	Cheng,T.	Tob.Control	2014			ii11
17	Journal Article	Czogala,J. et al.	Nicotine Tob.Res.	2014	16	6	655
18	Journal Article	Drummon d,M. B.et al.	Ann.Am.Thorac .Soc.	2014	11	2	236
19	Journal Article	Etter,J. F.	Addiction	2014	109	5	825
20	Journal Article	Etter,J. F.et al.	Eur.Respir.J.	2011	38	5	121 9
21	Journal Article	Etter,J. F.et al.	Tob.Control	2011	20	3	243
22	Journal Article	Etter,J. F.et al.	Addiction	2013	108	9	167 1
23	Journal Article	Farsalinos ,K. E. et al.	Nicotine Tob.Res.	2015	17	2	168
24	Journal Article	Farsalinos ,K. E. et al.	Ther.Adv.Drug Saf.	2014	5	2	67
25	Journal Article	Farsalinos ,K. E. et al.	Int.J.Environ.Re s.Public.Health.	2013	10	10	514 6
26	Journal Article	Farsalinos ,K. E. et al.	Toxicol.In.Vitro .	2014	28	5	101 6

27	Journal Article	Farsalinos ,K. E. et al.	Sci.Rep.	2014	4		413 3
28	Journal Article	Flouris,A. D. et al.	Inhal.Toxicol.	2013	25	2	91
29	Journal Article	Flouris,A. D. et al.	BMJ	2010	340		c31 1
30	Journal Article	Fuoco,F. C. et al.	Environ.Pollut.	2014	184		523
31	Journal Article	Goniewic z,M. L. et al.	Int.J.Drug Policy	2015			
32	Journal Article	Goniewic z,M. L. et al.	Addiction	2014	109	3	500
33	Journal Article	Goniewic z,M. L. et al.	Tob.Control	2014	23	2	133
34	Journal Article	Goniewic z,M. L. et al.	Nicotine Tob.Res.	2013	15	1	158
35	Journal Article	Goniewic z,M. L. et al.	Nicotine Tob.Res.	2015	17	2	256
36	Journal Article	Grana,R. et al.	Circulation	2014	129	19	197 2
37	Journal Article	Hahn,J. et al.	Tob Induc Dis.	2014	12	1	23
38	Journal Article	Hajek,P. et al.	Addiction	2014	109	11	180 1
39	Journal Article	Hajek,P. et al.	Nicotine Tob.Res.	2015	17	2	175
40	Journal Article	Ingebreth sen,B. J. et al.	Inhal.Toxicol.	2012	24	14	976
41	Journal Article	Kosmider ,L. et al.	Nicotine Tob.Res.	2014	16	10	131 9
42	Journal Article	Laugesen, M.		2008			

43	Journal Article	Lisko,J. G. et al.	Nicotine Tob.Res.	2015			
44	Journal Article	Manigrasso,M.et al.	Environ.Pollut.	2015	196		257
45	Journal Article	Manigrasso,M.et al.	Environ.Pollut.	2015	202		24
46	Journal Article	McAuley, T. R. et al.	Inhal.Toxicol.	2012	24	12	850
47	Journal Article	McCauley,L. et al.	Chest	2012	141	4	1110
48	Journal Article	Misra,M. et al	Int.J.Environ.Res.Public.Health.	2014	11	11	11325
49	Journal Article	Oh,A. Y. et al.	Laryngoscope	2014	124	12	2702
50	Journal Article	Orr,M. S.	Tob.Control	2014			ii18
51	Journal Article	Palazzolo, D. L.	Front.Public.Health.	2013	1		56
52	Journal Article	Paradise,J.	Yale J.Health.Policy.Law.Ethics	2013	13	2	326
53	Journal Article	Pellegrino,R. M. et al.	Ann.Ig.	2012	24	4	279
54	Journal Article	Romagna, G. et al.	Inhal.Toxicol.	2013	25	6	354
55	Journal Article	Schober, W. et al.	Int.J.Hyg.Environ.Health	2014	217	6	628
56	Journal Article	Schraufnagel,D. E.	Pediatr.Allergy Immunol.Pulmonol.	2015	28	1	2

57	Journal Article	Schroeder,M. J. et al.	Tob.Control	2014			ii30
58	Journal Article	Trtchounian,A. et al.	Nicotine Tob.Res.	2010	12	9	905
59	Journal Article	Vansickel,A. R. et al.	Cancer Epidemiol.Biomarkers Prev.	2010	19	8	1945
60	Journal Article	Vansickel,A. R. et al.	Nicotine Tob.Res.	2013	15	1	267
61	Journal Article	Wagener, T. L. et al.	Addiction	2012	107	9	1545
62	Journal Article	Williams, M. et al.	PLoS One	2013	8	3	e57987
63	Journal Article	Yan,X. S. et al.	Regul.Toxicol.Pharmacol.	2015	71	1	24
64	Journal Article	Hureaux,J. et al.	Thorax	2014	69	6	596
65	Journal Article	Vardavas, Constantine I. et al.	CHEST Journal	2012	141	6	1400
66	Journal Article	Westenberger,B.,J.		2009			
67	Journal Article	Zhang,Y. et al.	Nicotine Tob.Res.	2013	15	2	501
68	Journal Article	Farsalinos,K. E. et al.	Int.J.Environ.Res.Public.Health.	2015	12	4	3439
69	Report	U.S. Department of Health and Human Services		2014			

70	Journal Article	Hadwiger ,M. E. et al.	J.Chromatogr.A	2010	1217	48	754 7
71	Journal Article	Werley,M . S. et al.	Toxicology	2011	287	4200 7	76
72	Report	Rousseau x,C.		2014	45003 06576		
73	Journal Article	Lerner,C. A. et al.	PLoS One	2015	10	2	e01 167 32
74	Journal Article	Geiss,O. et al.	Int.J.Hyg.Enviro n.Health	2015	218	1	169
75	Journal Article	Famele,M . et al.	Nicotine Tob.Res.	2015	17	3	271
76	Journal Article	Wu,Q. et al.	PLoS One	2014	9	9	e10 834 2
77	Journal Article	Talih,S. et al.	Nicotine Tob.Res.	2015	17	2	150
78	Journal Article	Hutzler,C. et al.	Arch.Toxicol.	2014	88	7	129 5
79	Journal Article	Marini,S. et al.	Toxicol.Appl.Ph armacol.	2014	278	1	9
80	Journal Article	Ruprecht, A. A. et al.	Tumori	2014	100	1	e24
81	Journal Article	Kim,H. J. et al.	J.Chromatogr.A	2013	1291		48
82	Journal Article	Cameron, J. M. et al.	Tob.Control	2014	23	1	77

83	Journal Article	Flouris,A. D. et al.	Food Chem.Toxicol.	2012	50	10	3600
84	Journal Article	Schripp,T. et al.	Indoor Air	2013	23	1	25
85	Journal Article	Tayyarah, R. et al.	Regul.Toxicol.Pharmacol.	2014	70	3	704
86	Journal Article	Trehy,M. L. et al.	Journal of Liquid Chromatography & Related Technologies	2011	34	14	1442
87	Journal Article	Uchiyama,S. et al.	Anal.Sci.	2013	29	12	1219
88	Journal Article	Lim,H. H. et al.	Bull. Korean Chem. Soc.	2013	34	9	2691
89	Journal Article	Tierney,P. A. et al.	Tob.Control	2015			
90	Journal Article	Talih,S. et al.	Nicotine Tob.Res.	2015			
91	Journal Article	Jensen,R. P. et al.	N.Engl.J.Med.	2015	372	4	392
92	Report	Labstat International ULC		2015			
93	Journal Article	Scheffler, S. et al.	Int.J.Environ.Res.Public.Health.	2015	12	4	3915
94	Journal Article	Durmowicz,E. L. et al.	Tob.Control	2015			
95	Journal Article	Hubbs,A. F. et al.	Am.J.Pathol.	2012	181	3	829

96	Journal Article	Bullen,C. et al.	Tob.Control	2010	19	2	98
97	Web Page	Eco Vapour Canada		2015	2015	42124	
98	Journal Article	Suber,R. L. et al.	Food Chem.Toxicol.	1989	27	9	573
99	Journal Article	England, L. J. et al.	Am.J.Prev.Med.	2015			
100	Journal Article	Martinez, R. E. et al.	Nicotine Tob.Res.	2014			
101	Journal Article	Long,G. A.	Int.J.Environ.Res.Public.Health.	2014	11	11	11177
102	Journal Article	Colard,S. et al.	Int.J.Environ.Res.Public.Health.	2014	12	1	282
103	Journal Article	Lerner,C. A.et al.	Environ.Pollut.	2015	198		100
104	Journal Article	Vakkalanka,J. P.et al.	Clin.Toxicol.(Phila)	2014	52	5	542
105	Journal Article	Varlet,V.et al.	Int.J.Environ.Res.Public.Health.	2015	12	5	4796
106	Journal Article	O'Connell,G.et al.	Int.J.Environ.Res.Public.Health.	2015	12	5	4889
107	Journal Article	Sussan,T. E. et al.	PLoS One	2015	10	2	e0116861
108	Journal Article	Hecht,S. S. et al.	Nicotine Tob.Res.	2015	17	6	704

109	Journal Article	Hua,M. et al.	Tob.Control	2013	22	2	103
110	Journal Article	Etter,J. F. et al.	Addiction	2011	106	11	2017
111	Journal Article	Behar,R. Z. et al.	PLoS One	2015	10	2	e0117222
112	Journal Article	McRobbie,H. et al.	Cochrane Database Syst.Rev.	2014	12		CD010216
113	Journal Article	Zhu,S. H. et al.	Tob.Control	2014			iii3
114	Report	Government of Canada		2015			
115	Journal Article	Pierce,J. S. et al.	Crit.Rev.Toxicol.	2014	44	5	420
116	Journal Article	Dawkins, L. et al.	Addiction	2013	108	6	1115
117	Journal Article	Farsalinos,K. E. et al.	Int.J.Environ.Res.Public.Health.	2014	11	4	4356
118	Journal Article	Benowitz, N. L. et al.	Clin.Pharmacol. Ther.	1984	35	4	499
119	Journal Article	Laugesen, M.	N.Z.Med.J.	2015	128	1411	77
120	Journal Article	Spindle,T. R. et al.	Nicotine Tob.Res.	2015	17	2	142
121	Journal Article	Weaver, M. et al.	J.Addict.Med.	2014	8	4	234

122	Journal Article	Eissenberg,T.	Tob.Control	2010	19	1	87
123	Journal Article	Farsalinos,K. E. et al.	Addiction	2015			
124	Journal Article	Eissenberg,T. et al.	Nicotine Tob.Res.	2015	17	2	165
125	Journal Article	McGrath-Morrow,S. A. et al.	PLoS One	2015	10	2	e0118344
126	Journal Article	Thota,D. et al.	J.Emerg.Med.	2014	47	1	15
127	Journal Article	Lim,H. B. et al.	Toxicol.Res.	2014	30	1	13
128	Report	Rijksinstituut voor Volksgezondheid en Milieu (RIVM)		2015			
129	Web Page	ABC News		2015	2015	42156	
130	Web Page	The Jerusalem Post		2013	2015	42156	
131	Web Page	American Association of Poison Control Centres		2015	2015	42156	
132	Journal Article	Kavvalakis,M. P. et al.	J.Anal.Toxicol.	2015	39	4	262
133	Journal Article	Fowles,J. R. et al.	Crit.Rev.Toxicol.	2013	43	4	363
134	Journal Article	Agency for Toxic Substances and Disease Registry		2008			

135	Journal Article	Agency for Toxic Substances and Disease Registry (ATSDR)		1997			
136	Journal Article	Gaworski, C. L. et al.	Toxicology	2010	269	1	54
137	Report	Rijksinstituut voor Volksgezondheid en Milieu (RIVM)		2015			
138	Journal Article	Renne,R A. et al.	Inhal.Toxicol.	1992	4	2	95
139	Journal Article	Center for the Evaluation of Risks to Human Reproduction		2004	18	4	533
140	Journal Article	Robinson, R. J. et al.	PLoS One	2015	10	6	e0129296
141	Journal Article	Farsalinos,K. E. et al.	Sci.Rep.	2015	5		11269
142	Report	Rijksinstituut voor Volksgezondheid en Milieu (RIVM)		2015			
143	Journal Article	Rubenstein,D. A. et al.	Mol.Immunol.	2015			
144	Journal Article	Palpant,N. J. et al.	PLoS One	2015	10	5	e0126259

145	Journal Article	Lindgren, M. et al.	Psychopharmacology (Berl)	1999	145	3	342
146	Journal Article	Fishbein, L. et al.	J.Investig.Med.	2000	48	6	435
147	Journal Article	Meo,S. A. et al.	Eur.Rev.Med.Pharmacol.Sci.	2014	18	21	3315
148	Journal Article	Hua,M. et al.	J.Med.Internet Res.	2013	15	4	e59
149	Journal Article	Pisinger, C. et al.	Prev.Med.	2014	69		248
150	Journal Article	Etter,J. F. et al.	Addict.Behav.	2014	39	2	491
151	Journal Article	Kim,S. et al.	Int.J.Environ.Res.Public.Health.	2015	12	5	4859

Section 1: Open Characterization Project- Assigning roles to detected chemicals in vaping liquids

A wide variety of chemical compounds in vaping products have been detected in the Open Characterization Project. These chemicals have been assigned roles in order to have a better understanding of the part they may play within a vaping liquid. To identify the likely role(s) of each chemical compound, a literature synthesis was conducted which involved drawing from a variety of sources including published literature, open source websites (PubChem (National Institutes of Health (NIH) 2021a), Chem Spider ((Royal Society of Chemistry 2021)), The Human Metabolome Database (HMDB) (Wishart et al. 2018), Flavor DB (Garg et al. 2018), FooDB (Harrington et al. 2019)), manufacturer specifications, patents, Safety Data Sheets (SDS) and other. In addition, a number of chemical compounds are assigned roles based on an in-house database of chemicals which was developed between 2015 and 2017, namely eCigDB. This database of chemical compounds in vaping liquids was compiled from previously reported peer-reviewed studies and used to create a list of expected compounds in vaping products as well as increase confidence in the chemicals identified in the Open Characterization Project.

Chemicals are classified into at least one of the six (6) roles listed below. Each of the roles includes examples of supporting literature to help illustrate how roles were developed and assigned to the various chemicals.

Assigned roles for detected chemicals:

1.) Alkaloids - Class of organic compounds that contain a basic nitrogen atom. In vaping liquids, these most frequently include nicotine and nicotine-related minor alkaloids. Supporting literature used to

assign this role are published scientific studies from PubMed, data obtained from PubChem, and also the following data sources:

- The Chemical Components of Tobacco and Tobacco Smoke by Alan Rodgman, Thomas A. Perfetti (Second Edition)
- Chemistry of alkaloids by P.B. Saxena (2007)

2.) Processing - Various chemical compounds known to be used in the manufacture of vaping and tobacco products, flavours and food industry such as solvents, diluents, and processing agents. In addition to patents, PubChem, Chem Spider and other open sources the following resources were used:

- Good Scents website(Good Scents Company 2021)
- Leffingwell database (Leffingwell & Associates 2011)
- Manufacture's web pages e.g. The Perfumer's Apprentice(The Flavor and Perfumer's Apprentice 2021)
- Substances Added to Food (formerly Everything Added to Foods in the United States (EAFUS))(U.S. Food and Drug Administration 2021)
- Directories for chemical suppliers, which often lists fine chemicals and describes type of chemical synthesis e.g. LookChem

3.) Natural Extracts - Natural extracts are generally complex mixtures of individual chemical compounds previously detected in extracts of living organisms (examples include plants and animals). These chemical compounds may have organoleptic properties and are most frequently searched for on PubChem, Good Scents, FooDB, and HMDB. PubMed searches with these names and CAS numbers often yield examples of non-targeted studies on extracts or essential oils of various plants and where these compounds might be present.

4.) Flavours and Fragrances - Chemical compounds that have organoleptic/sensory properties and are known to impart flavour and/or odour. In most cases data sources are Leffingwell Flavor-Base, Good Scent Company, The Flavor and Extract Manufacturers Association (FEMA)((FEMA) 2021), Joint FAO/WHO Expert Committee on Food Additives (JECFA)(Food and Agriculture Organization of United Nations 2021) and Fenaroli's Handbook on Flavour Ingredients (Burdock 2016). In addition, online resources from suppliers of raw aromatic and flavour materials were used as well e.g. The Flavor and Perfumer's Apprentice(The Flavor and Perfumer's Apprentice 2021)

5.) Indirect Additive/Leaching/Degradation - Chemical compounds previously detected as indirect additives in food contact materials. This category also includes chemicals known to be associated with degradation of various other chemical compounds found to be present in the individual samples, for example, glycidol being a degradation product of glycerin. Sources of information include PubChem, Substances Added to Food (formerly EAFUS) and searching of published studies on degradation, indirect food contact additives and leaching of various components of vaping liquids.

6.) Unknown roles - Two types of chemical compounds are identified with unknown roles:

(1) Chemicals whose identities are not known. Chemicals whose identities are unknown are those that are true unknowns, meaning upon use of spectral library and spectral matching no appropriate chemical match was found in any mass spectral library used; National Institute of Standards and Technology (NIST), Wiley or Flavors and Fragrances of Natural and Synthetic Compounds. Since no International Union of Pure and Applied Chemistry (IUPAC) chemical name or

Chemical Abstracts Service (CAS) number could be identified for these chemical compounds their roles could not be determined.

(2) Chemicals for which identities are tentatively identified. There are a number of chemical compounds detected in vaping liquids for which identification is tentatively assigned (chemical name and CAS known) but for which literature synthesis generated none or inconclusive data. For example, a proportion of these chemical compounds with unknown role in vaping liquids did have matches with chemical compounds previously detected in yeast extract. Data matching was accomplished through publicly available informatics platform and data repository Yeast Resource Centre (University of Washington 2018). Yeast extract is a mixture of individual chemical compounds that may be used as food flavouring or enhancer(U.S. Food and Drug Administration 2020). Although a number of yeast related compounds detected in the Open Characterization Project could be originating from the yeast extract used for flavouring, some could be present as a result of product ageing, fermentation or presence of other microbes.

Chemicals with multiple roles

According to literature pertaining to the detected chemicals some may play multiple roles in vaping liquids, therefore more than one role may be assigned to a particular chemical. For example, a chemical may be found in a natural extract as well as have organoleptic properties, thus this chemical would be assigned the roles of natural extract and flavour. Literature used for assigning multiple roles have been discussed under various roles listed above.

Section 2: Open Characterization Project- Analytical method validation and performance

The analytical methods that employ the use of non-targeted analysis are most frequently full-scan methods that do not quantify but rather aim to identify chemical compounds present in the samples analyzed. In operating mass spectrometry detector in a full scan mode concentration levels are not determined, hence the limit of detection or ability to quantify a specific chemical at a prescribed concentration in a consistent manner is not an appropriate measure for method performance. Furthermore, the area under the detected peak for chemical generated in the full scan should not be relied upon to generate a corresponding calibrated concentration level. Instead validation can be performed using the repeated injection of the same sample over a period of time and checking for the detected compounds. In our case we have used laboratory prepared vaping liquid sample consisting of matrix (PG/VG in 50/50 w/w) and nicotine. This sample along with repeat analysis of previously analyzed sample was injected with processing batches or anytime maintenance was performed on the instruments. For example, rough estimates of signal to noise measurements while using full-scan were used for known, laboratory prepared nicotine concentration. Sample of laboratory-prepared vaping liquid containing nicotine, PG/VG and diluted 200 times with methanol were used to simulate and determine at which point nicotine would no longer be detected through varying added concentrations of nicotine and observing signal to noise after each injection. In case of nicotine, limit of detection using full scan was estimated using 3:1 signal to noise and determined at 0.03 mg/mL in this laboratory prepared vaping liquid sample. Such determination for all 1507 chemicals detected is not practical, needed, nor possible given the fact that genuine analytical standards for some infrequently detected chemicals are simply not available for commercial purchase. Further analytical efforts and investments in determining concentrations and limits of detection for non-prioritized compounds would not be justifiable.

Individual limits of detection for analytes of interest have been or will be determined employing the appropriate targeted methods which, depending on the compound, may include chemical extraction prior to analysis and use of labelled internal standards to correct for matrix effects. For example, targeted methodology for quantitation of nicotine has been already developed. Briefly this method employs use of single reaction monitoring (srm) mode while monitoring following ions: Nicotine (m/z) 84 and 162, quantifier and qualifier ions, respectively, Nicotine d7 (m/z) 87 and 169, quantifier and qualifier ions, respectively. Method

performance of this method was assessed according to the EPA Regulation 40 CFR part 136 (Appendix B) method (U.S. Environmental Protection Agency 2011). Eight replicates of laboratory prepared vaping liquid using USP grade PG, VG and fortified to a nicotine level of 0.05 mg/mL were put through sample extraction and analyzed. The standard deviation associated with eight replicate analyses of laboratory prepared vaping liquid and processed through the entire analytical procedure was multiplied by the Student's t value of 2.998 (appropriate for a 99% confidence level with 7 degrees of freedom). The method detection limit (MDL) for nicotine was calculated to be 0.002 mg/mL. The limit of quantitation (LOQ) was calculated according to the US EPA method, where the standard deviation associated with the eight replicate analyses of laboratory prepared vaping liquid conducted to obtain the MDL was multiplied by a factor of 10. The LOQ was calculated to be 0.006 mg/mL. The example of nicotine provided here, shows significant increase in detection limits through the use of targeted approach, hence, follow up, targeted methods will include subset of samples that through full scan have been found to contain identified analyte of interest and those that have not.

Another approach to validate non-targeted methodology in our case was through the use of two different GC MS/MS instruments. This idea of validation for non-targeted approach through the use of different instrumental platforms has been studied more extensively and at a larger scale through U.S. EPA's collaboration trial ENTACT (Ulrich et al. 2019) as well as NORMAN network collaborative trial in Europe (Schymanski et al. 2015). Briefly, in Open Characterization study, 15 random samples from various flavour profiles of vaping liquids were analyzed using both, 7000C and Quantum GC, instruments. Although many similar chemicals were reported by the both systems, certain compounds were more likely to be detected by one system than the other in the full scanning mode. On average, Quantum GC was able to detect higher number of compounds in the same product presumably due to the fact that it is a higher-end, more sensitive instrument. However, 7000C was able to detect more frequently, polycyclic aromatic hydrocarbons (PAHs) due to the fact that this instrument is also known as a PAH analyzer. The proportion of identical chemicals detected by the both systems for the same sample analyzed ranged between 41 and 83%, (average 55%). This percent of overlap is significantly higher compared to NORMAN study results of 5.4% overlap among participating laboratories as well as ENTACT trial's preliminary results of 2.9% overlap when comparing GC, LC electro-spray ionization (ESI-), and LC ESI+, based methodologies. Higher overlap in Open Characterization study is likely due to a number of factors. First, vaping liquids are chemically less complex compared to the tested samples in both collaboration trials, raw river water (NORMAN), and, house dust, serum and silicone bands (ENTACT). Moreover, our comparison is between two GC MS/MS systems, run in the same laboratory environment with minimal sample pre-treatment, dilution with solvent only. For ENTACT trial, for example, sample extraction and pre-treatment, as well as analytical instruments differed significantly among participating laboratories. Finally, in our study the list of expected or "suspect" chemicals compiled from published literature was identical for two instruments used.

In the end, in order to complete the project of this magnitude in a timely manner, 810 vaping samples were analyzed using only one of the two available instruments. Samples for each instrument were assigned randomly. In order to account for bias in the subsequent targeted analysis, samples with positive detection as well as those with negative will be analyzed.

Section 3: Open Characterization Project- Automated Mass Spectral Deconvolution and Identification System (AMDIS)

AMDIS is an open source software used to aid in interpretation of complex GC/MS generated spectral data. The process is automated where the software detects background traces, calculates automatically noise levels and analyzes data and ion traces to detect peak maxima and unique traces to yield a clean spectra that can be searched in a spectral library (Stein 1999).

An example of a more complex sample of Creamy Custard with labeled 18mg/mL nicotine and 60/40 (PG/VG proportion) is illustrated in Figure S1. AMDIS has been especially useful in identifying detected compounds that co-elute with a very broad glycerol peak such as this one Figure S2.

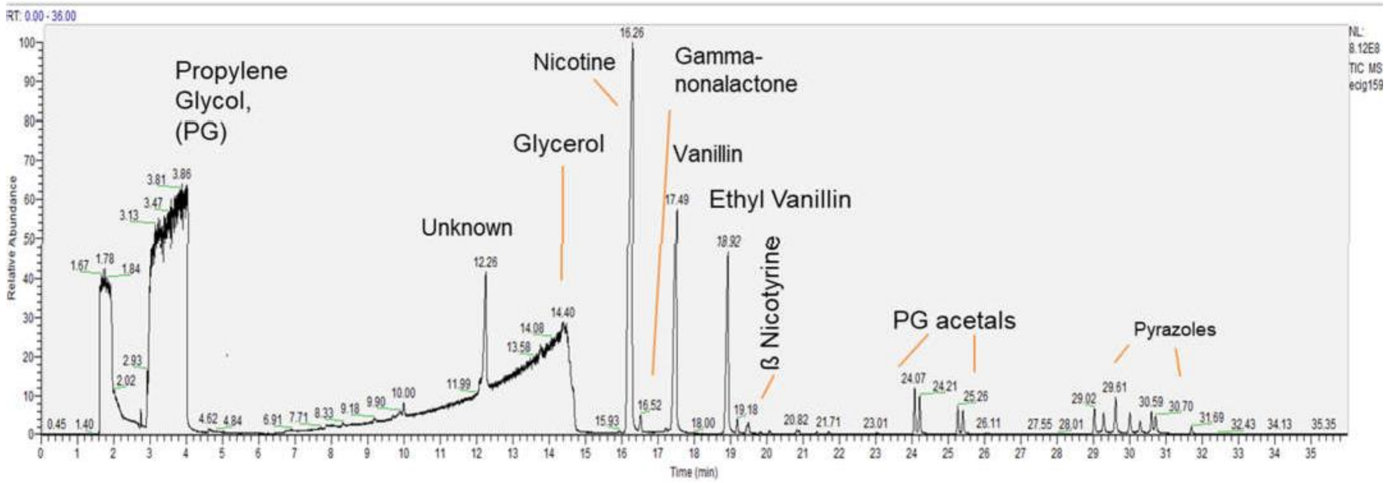

Figure S1. Chromatogram of Creamy Custard sample with co-eluting unknown peak

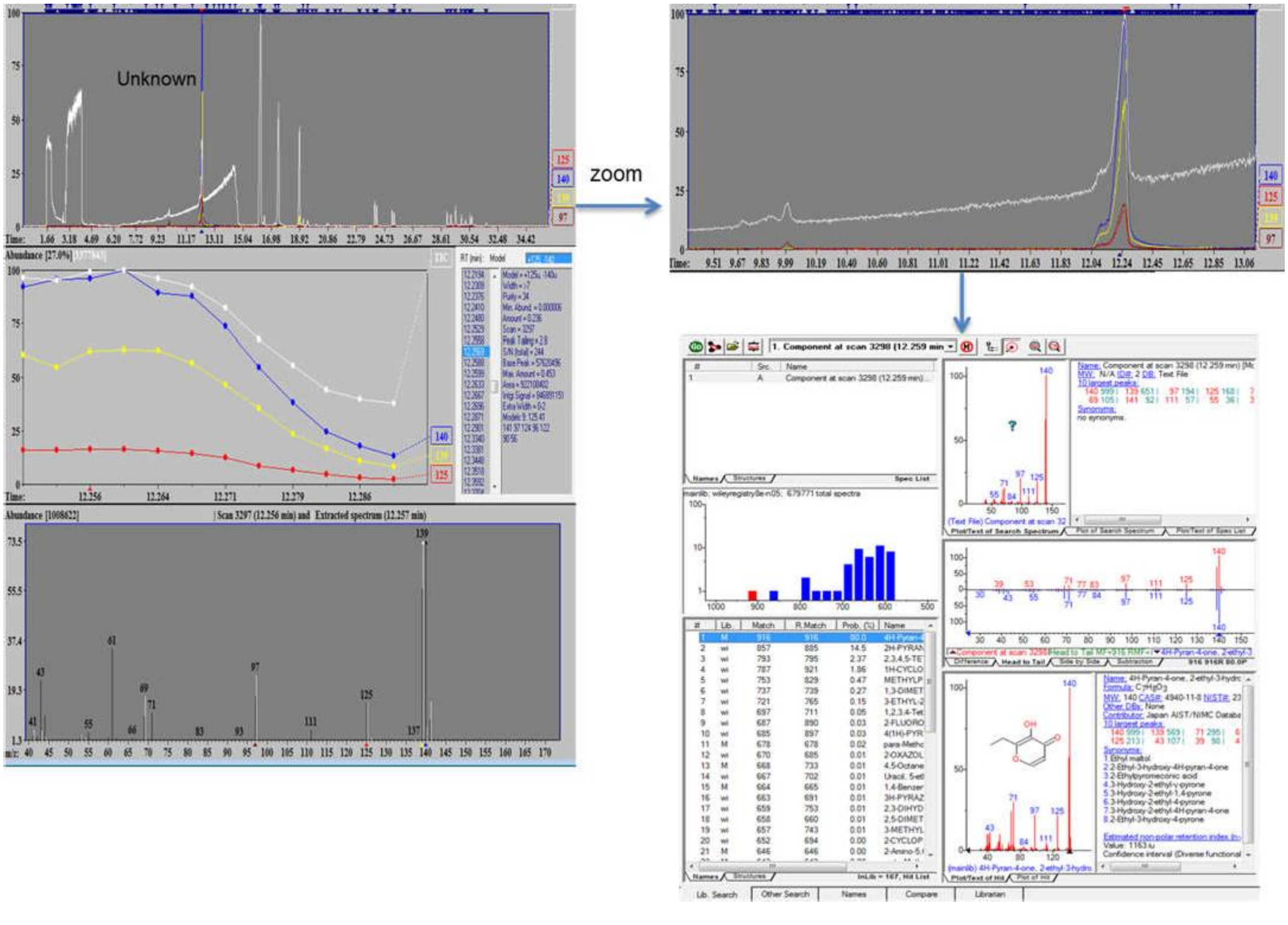

Figure S2. Example of AMDIS workflow for Creamy Custard sample

(FEMA), The Flavor and Extract Manufacturers Association of the United States. 2021. "FEMA Database." https://www.femaflavor.org/about. https://www.femaflavor.org/about.

Burdock, George A. 2016. *Fenaroli's handbook of flavor ingredients*. CRC press.

Food and Agriculture Organization of United Nations. 2021. Online Edition: "Combined Compendium of Food Additive Specifications".

Garg, Neelansh, Apuroop Sethupathy, Rudraksh Tuwani, Rakhi Nk, Shubham Dokania, Arvind Iyer, Ayushi Gupta, Shubhra Agrawal, Navjot Singh, and Shubham Shukla. 2018. "FlavorDB: a database of flavor molecules." *Nucleic acids research* 46 (D1): D1210-D1216.

Good Scents Company. 2021. "The Good Scents Company Information System." http://www.thegoodscentscompany.com/index.html.

Harrington, Richard Andrew, Vyas Adhikari, Mike Rayner, and Peter Scarborough. 2019. "Nutrient composition databases in the age of big data: foodDB, a comprehensive, real-time database infrastructure." *BMJ open* 9 (6): e026652.

Leffingwell & Associates. 2011. "Flavor-Base 10 - Tobacco Version. 2011." http://www.leffingwell.com/tob2001.htm.

National Institutes of Health (NIH). 2021a. "PubChem." https://pubchem.ncbi.nlm.nih.gov/.

Royal Society of Chemistry. 2021. "ChemSpider." http://www.chemspider.com/.

Schymanski, Emma L, Heinz P Singer, Jaroslav Slobodnik, Ildiko M Ipolyi, Peter Oswald, Martin Krauss, Tobias Schulze, Peter Haglund, Thomas Letzel, and Sylvia Grosse. 2015. "Non-target screening with high-resolution mass spectrometry: critical review using a collaborative trial on water analysis." *Analytical and bioanalytical chemistry* 407 (21): 6237-6255.

Stein, Stephen E. 1999. "An integrated method for spectrum extraction and compound identification from gas chromatography/mass spectrometry data." *Journal of the American Society for Mass Spectrometry* 10 (8): 770-781.

The Flavor and Perfumer's Apprentice. 2021. "The Flavor and Perfumer's Apprentice." https://shop.perfumersapprentice.com/.

U.S. Environmental Protection Agency. 2011. "40 CFR Appendix B to Part 136 - Definition and Procedure for the Determination of the Method Detection Limit-Revision 1.11." https://www.govinfo.gov/app/details/CFR-2011-title40-vol23/CFR-2011-title40-vol23-part136-appB.

U.S. Food and Drug Administration. 2020. Yeast-malt sprout extract. In *https://www.accessdata.fda.gov/scripts/cdrh/cfdocs/cfcfr/CFRSearch.cfm?fr=172.590*.

U.S. Food and Drug Administration. 2021. "Substances Added to Food." https://www.cfsanappsexternal.fda.gov/scripts/fdcc/?set=FoodSubstances

Ulrich, Elin M, Jon R Sobus, Christopher M Grulke, Ann M Richard, Seth R Newton, Mark J Strynar, Kamel Mansouri, and Antony J Williams. 2019. "EPA's non-targeted analysis collaborative trial (ENTACT): genesis, design, and initial findings." *Analytical and bioanalytical chemistry* 411 (4): 853-866.

University of Washington. 2018. "The Yeast Resource Center (YRC) ". http://depts.washington.edu/yeastrc/.

Wishart, David S, Yannick Djoumbou Feunang, Ana Marcu, An Chi Guo, Kevin Liang, Rosa Vázquez-Fresno, Tanvir Sajed, Daniel Johnson, Carin Li, and Naama Karu. 2018. "HMDB 4.0: the human metabolome database for 2018." *Nucleic acids research* 46 (D1): D608-D617.
